# Attacking cryptosystems by means of virus machines

**DOI:** 10.1038/s41598-023-49297-6

**Published:** 2023-12-09

**Authors:** Mario J. Pérez-Jiménez, Antonio Ramírez-de-Arellano, David Orellana-Martín

**Affiliations:** 1 Research Group on Natural Computing, Department of Computer Science and Artificial Intelligence, Universidad de Sevilla, Avda. Reina Mercedes s/n, 41012 Sevilla, Spain; 2 SCORE Laboratory, I3US, Universidad de Sevilla, Avda. Reina Mercedes s/n, 41012 Sevilla, Spain

**Keywords:** Computational science, Computer science, Information technology

## Abstract

The security that resides in the *public-key cryptosystems* relies on the presumed computational hardness of mathematical problems behind the systems themselves (e.g. the *semiprime factorization problem* in the **RSA** cryptosystem), that is because there is not known any polynomial time (classical) algorithm to solve them. The paper focuses on the computing paradigm of *virus machines* within the area of Unconventional Computing and Natural Computing. Virus machines, which incorporate concepts of virology and computer science, are considered as number computing devices with the environment. The paper designs a virus machine that solves a generalization of the semiprime factorization problem and verifies it formally.

## Introduction

In recent years, surpassing the computational capacity of today’s computers has become a major goal in the digital world. As a result, researchers have turned to unconventional models to revolutionize problem-solving approaches. *Natural computing*^1^, a branch of Unconventional computation inspired by what occurs in nature, has gained significant traction with the emergence of neural networks, cellular automata, membrane computing, etc.

In the vast field of virology, a virus stands as a diminutive yet potent infectious agent that crucially relies on infiltrating a host cell to perpetuate its reproductive cycle. With astonishing versatility, viruses showcase their ability to infiltrate not only animals but also plants and protist species, thus affirming their ubiquity within the intricate tapestry of the natural world. A staggering estimation postulates the coexistence of approximately 350 trillion viruses, a colossal quantity that surpasses the number of bacteria and cells by an astounding factor of 10. As the COVID-19 pandemic permeated society, the scientific community, as well as the populace at large, bore witness to the devastating capabilities harboured within the realm of viruses. Such pervasive prevalence inherently instigates an intellectual curiosity that beckons the exploration of the intricately woven computational aspects underlying this captivating biological structure, for more detail information we refer to^2^.

Within the branch of unconventional computing, a promising and young model called *virus machines*^3^ has garnered attention. Inspired by the movement and replication behaviour of viruses within *hosts*, virus machines utilize an instruction graph to navigate and replicate itself through different channels within a system. Virus Machines have been studied as function computing mode, recognizer mode, and generating mode.

This computing paradigm is universal (as powerful as turing machines) when there is no bounded restrictions on the number of hosts, instructions and viruses in the host^3^. In addition, it seems to be useful to solve mathematical problems in a numerical point of view, authors in^4^ computed an arithmetic calculator and two pairing functions by virus machines. Finally, authors in^5^ opened a computational complexity theory through these devices.

*Cryptography* is a scientific discipline that concerns the security of the information in the presence of possible intruders, as well as authentication and identification, providing privacy and integrity. There are cryptosystems where the issuer has both a public and a private key to encrypt and decrypt (called *asymmetrics*), within asymmetric cryptography, highlights the *public-key cryptosystems*. The first published public key cryptosystem, named **RSA**, was established by Rivest et al.^6^.

The paper is organized as follows. Next section briefly describes different versions of the integer factorization problems, and the next section to it is devoted to the **RSA** cryptosystem. In “Basic virus machines”, the computing paradigm of virus machines is presented. “Solving the least prime divisor problem by a deterministic virus machine” is devoted to describe a deterministic virus machine solving a generalization of the *semiprime factorization problem*. The paper ends with some open problems and concluding remarks.

## The integer factorization problems

The *integer factorization problem* is the following: *given a natural number greater than 1, find its prime factorization*. It is conjectured to be a computationally hard problem. A decision version of this problem is the semiprime factor problem that can be described as follows: *given two natural numbers x, y greater than 1, determine whether or not x has a factor less than y*. This problem belongs to the complexity classes **NP**, **co-NP** and **BPQ**^7^. It is interesting to notice that if that problem is **NP**-complete or **co-NP**-complete then **NP** $=$ **co-NP**, for further information we refer to^8^.

A generalization of the *semiprime factorization problem* is the *least prime divisor* (LPD): “Given a natural number $n \ge 2$, determine the least natural number $m \ge 2$ such that *m* is a divisor of *n*”. Obviously, such *m* must be a prime number. This problem can be characterized by the partial function $\mathtt{F_{LPD}}$ defined as follows: $\mathtt{F_{LPD}}(n)=m$ if and only if $n \ge 2$ and *m* is least divisor of *n* greater than 2. Throughout this paper, a deterministic virus machine solving the *least prime divisor problem* (that is, computing the partial function $F_{LPD}$), will be designed and the formal verification of it will be considered.

## The RSA cryptosystem

The **RSA** algorithm was the first public-key cryptosystem to verify the conditions formulated by Diffie and Hellman in its seminal work^9^. It was introduced by Rivest et al.^6^. The underlying presumed computationally hard problem of the **RSA** system is the *semiprime factorization problem*, previously described. This problem becomes unfeasible when very large semiprimes are considered as inputs. Consequently, decrypt the information provide by the **RSA** system is believed to be a computationally intractable problem as well as some hard problems used in cryptography (i.e. the *knapsack problem* in Merkle-Hellman cryptosystem^10^). In fact, it has not been proven that the existence of an efficient algorithm for factoring large semiprimes is not possible. Nevertheless, some pathways have been studied with other unconventional computing models, such as quantum computation, which highlights recent works in the benefits of quantum communication or the combination of quantum communication with classical cryptography^11,12^.

Any large semiprime input *n* for **RSA** is used as the *modulus* for both public and private keys. In order to attack the **RSA** system, the factorization of *n* is needed. Many systems, such as banks, medical databases and critical systems with confidential information, keep their data secure thanks to this method. It is interesting to highlight the inherent asymmetry between generating large semiprimes and factoring them.

## Basic virus machines

This young computing paradigm, inspired by the way viruses are transmitted and replicated between hosts, was first introduced in^3^. Next, the **syntax** of this paradigm is formally defined.

### Definition 1

A virus machine (VM, for short) of degree (*p*, *q*), $p,q \ge 1$, is defined as the following tuple $\Pi =(\Gamma , H, I , D_H,D_I,G_C, n_1, \dots ,n_p, i_1,h_{out})$, where:

- $\Gamma =\{v\}$ is the singleton alphabet:
- $$H=\{h_1,\dots ,h_p\}$$ and $$I=\{i_1,\dots ,i_q\}$$ are ordered sets such that $$v \notin H \cup I$$, $$H \cap I=\emptyset $$ and $$h_{out} \notin I \cup \{v\}$$: either $$h_{out} \in H$$ or $$h_{out} \notin H$$ (in this case, $$h_{out}$$ represents the environment and it is denoted by $$h_0)$$;
- $D_H=(H \cup \{h_{out}\},E_H, w_H)$ is a weighted directed graph (WDG for short), where $E_H \subseteq H \times (H \cup \{ h_{out} \})$ such that $(h,h) \notin E_H$, for each $h \in H$, out-degree$(h_{out})=$ 0, and $w_H$ is a mapping from $E_H$ onto $\mathbb {N}\setminus \{0\}$;
- $D_I=(I,E_I,w_I)$ is a WDG, where $E_I \subseteq I \times I$, $w_I$ is a mapping from $E_I$ onto $\mathbb {N}\setminus \{0\}$ and the out-degree of each node is zero, one or two;
- $G_C=(V_C,E_C)$ is an undirected bipartite graph, where $V_C= I \cup E_H$, being $\{I, E_H\}$ the partition associated with it, in addition each edge connect an element from *I* with, at most, an arc from $E_H$;
- $i_1 \in I$ and $n_j \in \mathbb {N}$, for each *j*, $1 \le j \le p$, where $\mathbb {N}$ is the set of natural numbers.

Formally, a *virus machine* $\Pi =(\Gamma , H, I , D_H,D_I,G_C, n_1, \dots ,n_p, i_1,h_{out})$ of degree (*p*, *q*), is a tuple that can be viewed as an ordered set of *p* *hosts* labelled as $h_1,\dots ,h_p$, where each host $h_j$ initially contains $n_j$ *viruses*, and an ordered set of *q* *control instruction units* labelled with $i_1,\dots ,i_q\in I$. In this work, symbol $h_{out}\notin H$ and it represents the *output region*, denoted by $h_0$ in this work. Arcs $(h_s, h_{s'})$, where $s \ne s'$, from the weighted directed graph $D_H$ represent *transmission channels* through which viruses can transmit from one host $h_s \in H$ to another different host or region $h_{s'}\in H \cup \{h_{0}\}$. The computation of a virus machine starts with the activation of instruction $i_1$. At any moment, at most one instruction $i_j$ is *activated*: if a instruction $i_j$ is attached to the channel $(h_s,h_{s'})$ with weight $w_{s,s'}$ and it is activated at instant *t*, then it will be *opened* and one virus will be transmitted and replicated $w_{s,s'}$ from $h_s$ to $h_{s'}$, so one virus is consumed at $h_s$, and $w_{s,s'}$ viruses reach $h_{s'}$. Unless this situation happen, every channel is *closed*.

Arcs from the directed graph $D_I$ represent the *instruction paths* that will follow the computation, and they have associated with a weight. Finally, the undirected bipartite graph $G_C$ represents the *instruction-channel relation* by which an edge $\{i_j, (h_s,h_{s'})\}$ indicates a control between instruction $i_j$ and channel $(h_s,h_{s'})$.

**Figure 1:**
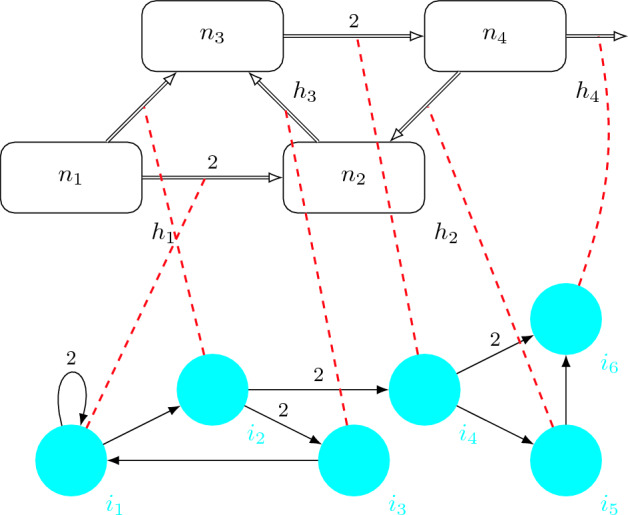
Structure of a $$virus \, machine$$.

Graphically, an example of virus machine of degree (4, 6), can be viewed in Fig. 1. As in previous works, each host is depicted as a rectangle and each instruction is drawn as a blue circle. The directed arrows are assigned with a *weight* (or weight 1 if it is not marked). The instruction-channel arcs are represented as dotted red lines.

The **semantics** are then described as follows: a *configuration* $\mathcal{C}_t$ or an *instant description* at an instant *t* of a virus machine is a tuple $(b_{1,t}, \dots ,b_{p,t}, u_t, b_{0,t})$ where $b_{0,t}, b_{1,t}, \dots ,b_{p,t}$ are natural numbers (representing the number of viruses within the environment $h_0$ and the hosts $h_1, \ldots , h_p$, respectively), and $u_t \in I \cup \{ \, \# \, \}$, being $\# \notin H \cup \{ h_0\} \cup I$. If $u_t \in I$, then $u_t$ will be activated at step $t+1$, otherwise, if $u_t=\#$, then no instruction will be activated and a halting configuration is reached. The *initial configuration* of a system $\Pi =(\Gamma , H, I , D_H,D_I,G_C, n_1, \dots ,n_p, i_1, h_{out})$ is defined as $\mathcal{C}_0=( n_1, \dots ,n_p, i_1,0)$. We say that a non halting configuration $\mathcal {C}_t=( b_{1,t}, \dots ,b_{p,t}, u_t,b_{0,t})$ yields configuration $\mathcal {C}_{t+1}=( b_{1,t+1}, \dots ,b_{p,t+1}, u_{t+1},b_{0,t+1})$ in one *transition step*, denoted by $\mathcal {C}_t \Rightarrow _{\Pi } \mathcal {C}_{t+1}$, if we can go to it in the following way.

- Assuming that the control instruction unit $u_t$ is attached to a channel $(h_s,h_{s'})$. If $b_{s,t} \ge 1$ then $b_{s+1,t}=b_{s,t}-1$ and $b_{s'+1,t}=b_{s',t}+w_{s,s'}$ If $b_{s,t} =0$ then $b_{s+1,t}=b_{s,t}$ and $b_{s'+1,t}=b_{s',t}$. If $u_t$ is not attached to any channel then there is no virus transmission.
- Object $u_{t+1} \in I \cup \{ \# \}$ is obtained depending on the *out*-*degree* of $u_t$ as follows:
  If *out*-*degree* of $(u_t)$ is equal to 2 and $(u_t,u_{t'}),(u_t, u_{t''} ) \in E_I$, with $t \ne t'$, then the following holds: (1) if $u_t$ is not attached to any channel then the next instruction $u_{t+1}$ will be either $u_{t'}$ or $u_{t''}$, chosen in a non-deterministic way; (2) if $u _t$ is attached to a channel $(h_s,h_{s'})$ and $b_{s,t} \ge 1$, then $u_{t+1}$ is maximum between $w_{t,t'}$ and $w_{t,t''}$, otherwise $u_{t+1}$ is minimum between these two instructions. In both cases, when $w_{t,t'}=w_{t,t''}$, either $u_{t+1}=u_{t'}$ or $u_{t+1}=u_{t''}$, are selected non-deterministically.
  If *out*-$degree (u_t)=1$ then the system behaves deterministically and $u_{t+1}$ follows the unic possible path in $E_I$.
  If there is no possible path, then $u_{t+1}=\#$, and $\mathcal{C}_{t+1}$ is a halting configuration.

A *computation* of a virus machine is a (finite or infinite) sequence of configurations such that: (a) the first term is the initial configuration $\mathcal{C}_0$ of the system; (b) for each $n \ge 2$, the *n*-th term of the sequence is obtained from the previous term in one transition step; and (c) if it is a *halting computation* (the sequence is finite) then the last term is a halting configuration.

### *Remark 1*

Virus machines are *sequential* devices, that is, in any instant at most a control instruction unit is activated and, hence, at most one transmission channel will be opened.

### Definition 2

A virus machine with input, of degree $(p,q,r), p \ge 1, q \ge 1, r \ge 1,$ is a tuple $\Pi =(\Gamma , H, H_r, I , D_H,D_I,G_C, n_1, \dots ,n_p, i_1,h_{out}),$ where:

- $(\Gamma , H, I, D_H,D_I,G_C, n_1, \dots ,n_p, i_1,h_{out})$ is a Virus Machine of degree (*p*, *q*).
- $H_r= \{h_{a_1}, \dots ,h_{a_r}\} \subseteq H$ is the ordered set of *r* input hosts and $h_{out} \notin H_r$.

If $\Pi$ is a virus machine with input of degree (*p*, *q*, *r*) and $(\alpha _1, \dots , \alpha _r) \in \mathbb {N}^r$, the *initial configuration* of $\Pi$ *with input* $(\alpha _1, \dots , \alpha _r)$ is $( n_1, \dots , n_{a_1}+\alpha _1, \dots , n_{a_r}+\alpha _r, \dots , n_p, i_t, 0)$, denoted by $\Pi + (\alpha _1,\dots , \alpha _r)$ proceeds as stated before. The result of a halting computation is the number of viruses sent to the output region (the environment) during that computation.

Next, virus machines providing function computing devices, is introduced.

### Definition 3

We say that a partial function $f : \mathbb {N}^k - \rightarrow \mathbb {N}$, $k\ge 1$. is computed by a virus machine $\Pi$ with *k* input hosts, if for each $(x_1, \dots ,x_k ) \in \mathbb {N}^k$, we have:

- If $(x_1, \dots ,x_k ) \in \text {dom}(f)$ with $f(x_1, \dots ,x_k )=z$, then every computation of the virus machine $\Pi + (x_1, \dots ,x_k )$ is a halting computation and the result is *z*.
- If $(x_1, \dots ,x_k ) \in \mathbb {N}\setminus \text {dom}(f)$, then every computation of the virus machine $\Pi + (x_1, \dots ,x_k )$ is a non-halting computation.

### Example of a deterministic virus machine

For a better understanding in the behavior of these devices, a basic example from^5^, is presented. Let $\Pi _{add}$ a deterministic virus machine computing the addition function $add:\mathbb {N}^2\rightarrow \mathbb {N}$, with $add(a,b) = a+b$, defined as follows:

$$\Pi =(\Gamma , H, H_2, I, D_H, D_I, G_C,0,0,i_1,h_0)$$, where:

- $$\Gamma = \{v\}$$, $$H = \{h_1,h_2\}=H_2$$, and $$I = \{i_1,i_2,i_3\}$$;
- $$D_H = (H,E_H = \{(h_1,h_0),(h_2,h_0)\},w_H)$$, where $$w_H(h_1,h_0)= w_H(h_1,h_0) = 1$$;
- $$D_I = (I, E_I = \{(i_1,i_1),(i_1,i_2),(i_2,i_2),(i_2,i_3)\},w_I)$$, where $$w_I(i_1,i_1)= w_I(i_2,i_2)= 2$$, and $$w_I(i_1,i_2)= w_I(i_2,i_3)= 1$$;
- $$G_C = (I\cup E_H, E_C = \{(i_1,(h_1,h_0)),(i_2,(h_2,h_0))\})$$

**Figure 2:**
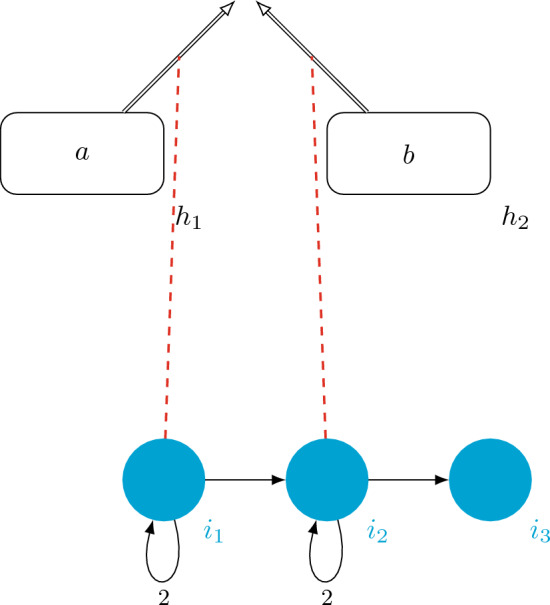
A *deterministic virus machine* $\Pi _{add}+(a,b)$ computing the addition function.

The VM $\Pi _{add}$, can be depicted in Fig. 2.

Given an input $(a,b)\in \mathbb {N}^2$, the computation of the machine woks as follows. The initial configuration is of $\Pi _{add}+(a,b)$, is $C_0=(0+a,0+b,i_1,0)$, from here, instruction $i_1$ opens the channel $(h_1,h_0)$, then the computation depends on the value of *a*:

- if $a> 0$, a virus is transmitted from host $h_1$ to the environment and the next instruction follows the highest weight path from $i_1$, that is going to $i_1$ again with the following configuration $C_1 =(a-1,b-1,i_1,1)$, instruction $i_1$ will be activated until host $h_1$ is empty, thus the configuration $C_a=(a-a,b,i_1,a)$ is reached.
- If $a=0$, then there is no virus transmission and next instruction is the least weight path, that is instruction $i_2$ and the following configuration remains, $C_1=(0,b,i_2,0)$.

Note that for all values of *a*, the configuration is $C_{a+1}=(0,b,i_2,a)$, from here, the computation is analogous and configuration $C_{a+1+b}=(0,b-b,i_2,a+b)$, as the host $h_2$ is now empty, there is no virus transmission and next instruction is $i_3$ as it follows the least weight path taking the configuration $C_{a+b+2}=(0,0,i_3,a+b)$, as $i_3$ is not attached to any channel and the $out-degree(i_3)$ is zero, we reach a halting configuration $C_{a+b+3}= (0,0,\#,a+b)$. That is, after $a+b+3$ steps, the machine halts and returns $a+b$, which is the addition of *a* and *b*.

## Solving the least prime divisor problem by a deterministic virus machine

In order to solve the *least prime divisor* problem by using virus machines, it suffices to design such a machine that computes the partial function $F_\texttt{LPD}$ characterizing this problem. Specifically, in this Section a deterministic virus machine that computes $F_\texttt{LPD}$, is presented.

From now on, since the description of a single virus machine can be tedious to read, it is easy to depict them with graphics and setting that $i_{1}$ is the initial instruction.

**Figure 3:**
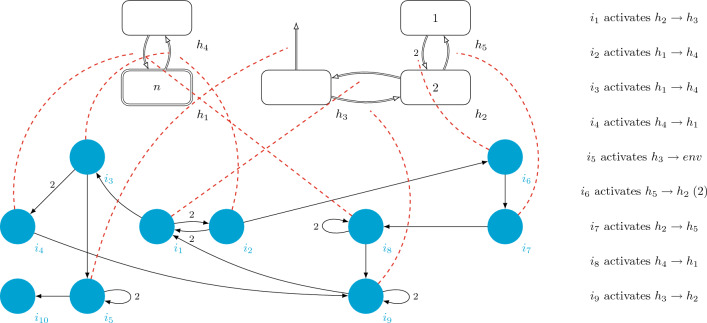
A *deterministic virus machine* $\Pi _{LPD}+n$ solving the LPD problem.

Let us consider the VM $\Pi _{LPD}=(\Gamma , H, H_1, I ,D_H,D_I,G_C,0,2,0,0,1, i_1,h_0)$ with input of degree (5, 10, 1), graphically defined in Fig. 3.

### A sketch of the formal verification

A method to formally verify that a computational device of a model solves a given problem is to find invariant formulas in some relevant loops of the device, in such a way that the veracity of those formulas at the end of the loops provides relevant information.

Next, we give a sketch of a virus machine with respect to the *least prime divisor* problem, that is, we must prove mathematically that the designed virus machine computes the partial function $F_\texttt{LPD}$ associated with that problem. Specifically, we will set the result an interesting input: for any odd number. For a detailed formal verification we refer to Appendix A.

Let us suppose that $n= p_1 \cdot q_{p_1}$ where $p_1\ne 2$ is the least prime factor and $q_{p_1}=\displaystyle \frac{n}{p_1}$. Let us consider the following notation:

1. For every $j\in \mathbb {N}$, such that $j\ge 2$, we consider $q_j$, the quotient of the integer division between *n* and *j*, and $\alpha _j = 4q_j+4n+7$.
2. Let $\beta _k = \alpha _2 + \dots + \alpha _k$, for every natural number $k\ge 2$.

Finally, let us consider the following formula:

$$\begin{aligned} \phi (k) \equiv C_{\beta _k} = (n,k+1,0,0,1,i_1,0), \text { for }2\le k \le p_1-1 \end{aligned}$$

This formula is an invariant of the machine, and by induction we get the following proposition.

#### Proposition 1

$$\forall k \, (\ 0 \le k \le p_1 -1 \longrightarrow \varphi (k) \ \text{ is } \text{ true } \, )$$.

From Proposition 1, we deduce that the formula $\phi (p_1-1)$ is true, thus we have $\phi (p_1-1)\equiv C_{\beta _{(p_1-1)}}=(\, n, \, p_1, \, 0, \, 0, \, 1,\, i_1, \, 0 \,)$, from here a halting configuration is reached after $q_{p_1}\cdot (3p_1+4)$ transition steps and returns $p_1$.

### Time complexity

Formal verification also results in a useful tool for studying the time complexity of a device as we know exactly the number of the steps necessary for the machine halts. From Remark 4 in Appendix A, we can conclude that the time complexity of this machine belongs to the quadratic order $O(n^2)$, for 1-ary encoding. The idea of the proof is to focus on the worst case, that is, when the input *n* is a prime number and to show that it is of quadratic order.

## Conclusions

The security of the first public-key cryptosystem (**RSA**) lies in the presumed intractability of the semiprime problem, from a computational complexity point of view. This problem is characterized by the partial function $\mathtt{F_{LPD}}$ defined as follows: $\mathtt{F_{LPD}}(n)=m$ if and only if $n \ge 2$ and *m* is least prime divisor of *n*. Throughout this paper, a deterministic virus machine solving the *least prime divisor*, has been designed and the formal verification of it is proved. Despite of the lack of efficiency that should be improved, these devices gives an interesting scope for solving numerical problems thanks to the mathematical integrity of the formal verification and its easy study of time complexity.

Furthermore, while the concept of virus machines is relatively new, there are numerous unexplored avenues within this field. Currently, the lack of efficiency of virus machines is because of its sequential behavior, and exploring parallelism could potentially enhance this aspect. Future research directions could involve expanding the model to incorporate additional biological aspects, deepening our understanding of virus functionality and exploring potential applications. Establishing connections between virus machines and traditional virology is also an intriguing area for exploration. Finally, while improvements in efficiency are studied, we believe that a specific hardware implementation could figure out an innovative scope.

### Supplementary Information

Supplementary Information.

## Data Availability

The datasets used and/or analysed during the current study available from the corresponding author on reasonable request.
