# Supplementary material for: Attacking cryptosystems by means of virus machines
Source: Sci Rep. 2023 Dec 9;13:21831. doi: 10.1038/s41598-023-49297-6 (PMC10710511; doi:10.1038/s41598-023-49297-6)
Supplement: Supplementary file 1 — Supplementary Information. [file 41598_2023_49297_MOESM1_ESM.pdf]

A Detailed formal verification

In this Appendix, a detailed formal verification of the Virus Machine Π_{LPD} is presented.

A.1 Any even natural number greater than 0

The computation of the virus machine Π with input a natural number $n = 2 \cdot t$, for each $t \geq 1$, is analyzed.

First of all, the result for $n = 2$ is shown by describing a trace of the computation:

$$
\begin{array}{lcl|lcl}
C_0 & = & (2, 2, 0, 0, 1, i_1, 0) & C_6 & = & (0, 0, 2, 2, 1, i_5, 0) \\
C_1 & = & (2, 1, 1, 0, 1, i_2, 0) & C_7 & = & (0, 0, 1, 2, 1, i_5, 1) \\
C_2 & = & (1, 1, 1, 1, 1, i_1, 0) & C_8 & = & (0, 0, 0, 2, 1, i_5, 2) \\
C_3 & = & (1, 0, 2, 1, 1, i_2, 0) & C_9 & = & (0, 0, 0, 2, 1, i_{10}, 2) \\
C_4 & = & (0, 0, 2, 2, 1, i_1, 0) & C_{10} & = & (0, 0, 0, 2, 1, \#, 2) \\
C_5 & = & (0, 0, 2, 2, 1, i_3, 0) & & &
\end{array}
$$

Therefore, in the case $n = 2$, the computation of the virus machine halts in, exactly, 10 transition steps, and the result of it is 2.

In the case n even and $n \geq 4$, let us consider the following formula:

$$\varphi(k) \equiv C_{10k} = (n - 2k, 2, 0, 2k, 1, i_1, 0)\ ,\ \text{for } 0 \leq k \leq \frac{n}{2} - 1$$

Proposition 2. $\forall k\,(\,0 \leq k \leq \frac{n}{2} - 1 \longrightarrow \varphi(k) \textit{ is true}\,)$.

Proof. By induction on k.
The base case $k = 0$ is trivial, because $C_0 = (n, 2, 0, 0, 1, i_1, 0)$.
Let k be such that $0 \leq k < \frac{n}{2} - 1$ and let us assume that $\varphi(k)$ is true. Let us see that $\varphi(k+1)$ is also true. Indeed, we have:

$$
\begin{array}{lcl|lcl}
C_{10k} & = & (n-2k, 2, 0, 2k, 1, i_1, 0) & C_{10k+6} & = & (n-2k-3, 0, 2, 2k+3, 1, i_4, 0) \\
C_{10k+1} & = & (n-2k, 1, 1, 2k, 1, i_2, 0) & C_{10k+7} & = & (n-2k-2, 0, 2, 2k+2, 1, i_9, 0) \\
C_{10k+2} & = & (n-2k-1, 1, 1, 2k+1, 1, i_1, 0) & C_{10k+8} & = & (n-2k-2, 1, 1, 2k+2, 1, i_9, 0) \\
C_{10k+3} & = & (n-2k-1, 0, 2, 2k+1, 1, i_2, 0) & C_{10k+9} & = & (n-2k-2, 2, 0, 2k+2, 1, i_9, 0) \\
C_{10k+4} & = & (n-2k-2, 0, 2, 2k+2, 1, i_1, 0) & C_{10k+10} & = & (n-2k-2, 2, 0, 2k+2, 1, i_1, 0) \\
C_{10k+5} & = & (n-2k-2, 0, 2, 2k+2, 1, i_3, 0) & & &
\end{array}
$$

That is, $C_{10(k+1)} = (n - 2(k+1), 2, 0, 2(k+1), 1, i_1, 0)$. Thus, $\varphi(k+1)$ is true.

□

From this Proposition, $\varphi(\frac{n}{2})$ is true. Bearing in mind that, in this case: $\frac{n}{2} - 1 = t - 1$, we have $C_{10(\frac{n}{2}-1)} = (2, 2, 0, 2(t - 1), 1, i_1, 0)$, that is, $C_{10(t-1)} = (2, 2, 0, n-2, 1, i_1, 0) = C_{5n-10}$. Therefore,

$$
\begin{array}{lcl|lcl}
C_{5n-9} & = & (2, 1, 1, n-2, 1, i_2, 0) & C_{5n-4} & = & (0, 0, 2, n, 1, i_5, 0) \\
C_{5n-8} & = & (1, 1, 1, n-1, 1, i_1, 0) & C_{5n-3} & = & (0, 0, 1, n, 1, i_5, 1) \\
C_{5n-7} & = & (1, 0, 2, n-1, 1, i_2, 0) & C_{5n-2} & = & (0, 0, 0, n, 1, i_5, 2) \\
C_{5n-6} & = & (0, 0, 2, n, 1, i_1, 0) & C_{5n-1} & = & (0, 0, 0, n, 1, i_{10}, 2) \\
C_{5n-5} & = & (0, 0, 2, n, 1, i_3, 0) & C_{5n} & = & (0, 0, 0, n, 1, \#, 2)
\end{array}
$$

Therefore, in the case n even and $n \geq 4$, the computation of the virus machine halts in, exactly, $5n$ transition steps, and the result of it is 2.

A.2 Any odd natural number greater than 1

Let us suppose that n is an odd number. We will use the notation from Section 5.1:

1. For every $j \in \mathbb{N}$, such that $j \geq 2$, we consider:

   $$\alpha_j = 4q_j + 4n + 7$$

   where q_j is the quotient of the integer division, i.e. $q_j = \lfloor \frac{n}{j} \rfloor$.

2. Let $\beta_k = \alpha_2 + \cdots + \alpha_k$, for every natural number $k \geq 2$.

3. Let $n = p_1 \cdot q_{p_1}$ where p_1 is the minimum prime factor and $q_{p_1} = \dfrac{n}{p_1}$

Let us remember the following formula

$$\phi(k) \equiv C_{\alpha_2+\alpha_1+\cdots+\alpha_k} = (n, k+1, 0, 0, 1, i_1, 0),\ \text{for } 2 \leq k \leq p_1 - 1$$

In this case, we focus on proving the invariance of this formula. For that, let us consider the following Lemma.

Lemma 3. *Let x, d natural numbers. Suppose that the configuration C_x of the virus machine, after x steps the computation is $C_x = (n, d, 0, 0, 1, i_1, 0)$, with $2 \le d \le p_1$. Then, for each natural number m such that $0 \le m \le q_d$, the following formula is verified*

$$C_{x+m(3d+4)} = (n - d\cdot m, d, 0, d\cdot m, 1, i_1, 0).$$

Except for $d = p_1$, in that case the formula is verified for each natural number m such that $0 \le m \le q_d - 1$.

Proof. We prove it by induction on m.

The base case $m = 0$ it is trivial, because $C_x = (n, d, 0, 0, 1, i_1, 0)$ by the hypothesis of the lemma.

Let m be such that $0 \le m < q_d$ and let us assume that the formula is verified. Let us see that it is also true for $m+1$, by induction hypothesis we have

$$C_{x+m(3d+4)} = (n - d\cdot m, d, 0, d\cdot m, 1, i_1, 0),$$

$$\begin{cases} C_{x+m(3d+4)+1} = (n-dm, d-1, 1, dm, 1, i_2, 0), \text{ as } h_2(d) \wedge d > 0 \\ C_{x+m(3d+4)+2} = (n-dm-1, d-1, 1, dm+1, 1, i_1, 0), \text{ as } h_1(n) \end{cases}$$

$$\ldots$$

$$C_{x+m(3d+4)+2d} = (n-dm-d, d-d, d, dm+d, 1, i_1, 0),$$

$$C_{x+m(3d+4)+2d+1} = (n-d(m+1), 0, d, d(m+1), 1, i_3, 0), \text{ as } h_2(0)$$

$$C_{x+m(3d+4)+2d+2} = (n-d(m+1)-1, 0, d, d(m+1)+1, 1, i_4, 0), \text{ as } h_1(n-d(m+1))$$

Note that for $d = p_1$, and for $m = q_{p_1} - 1 < q_d$, we do not have $n > d(m+1)$, instead, the result is the equality, so for the particular case of $d = p_1$, we will suppose that $m < q_1 - 1$.

$$C_{x+m(3d+4)+2d+3} = (n-d(m+1), 0, d, d(m+1), 1, i_9, 0),$$

$$\begin{cases} C_{x+m(3d+4)+2d+3+1} = (n-d(m+1), 1, d-1, d(m+1), 1, i_9, 0), \text{ as } h_3(d) \\ \ldots \\ C_{x+m(3d+4)+2d+3+d} = (n-d(m+1), d, d-d, d(m+1), 1, i_9, 0), \end{cases}$$

$$C_{x+m(3d+4)+3d+4} = (n-d(m+1), d, 0, d(m+1), 1, i_1, 0), \text{ as } h_3(0)$$

$$C_{x+(m+1)(3d+4)} = (n-d(m+1), d, 0, d(m+1), 1, i_1, 0)$$

Therefore, the result is verified for $m+1$. □

Remark 2. *If we consider the formula*

$$\mu_d(m) \equiv C_{x+m(3d+4)} = (n - d\cdot m, d, 0, d\cdot m, 1, i_1, 0)$$

for $0 \le m \le q_d$ and $2 \le d \le p_1$, then the result of the previous lemma establish the invariance of this formula (except for the particular case $m = q_d \wedge d = p_1$ noticed above).

Theorem 4. $\forall k$ *($2 \le k \le p_1 - 1 \to \phi(k)$ is true).*

Proof. In the base case $k = 2$, the computation starts in:

$$C_0 = (n, 2, 0, 0, 1, i_1, 0),$$

which satisfies the hypotesis of the Lemma 3 for $x = 0$ and $d = 2$, so $\mu_d(q_2)$ is true:

$$C_{q_2(3\cdot 2+4)} = (n - 2\cdot q_2, 2, 0, 2\cdot q_2, 1, i_1, 0),$$

Notice that $n - 2q_2 = r_2 = 1$ because n is an odd number.

$$\begin{array}{lcl|lcl} C_{q_2(3\cdot 2+4)} & = & (1,2,0,2\cdot q_2,1,i_1,0), & C_{10q_2+6+1} & = & (1,1,2,n-1,1,i_8,0), \\ C_{10q_2+1} & = & (1,2-1,1,2\cdot q_2,1,i_2,0), & & \ldots & \\ C_{10q_2+2} & = & (1-1,1,1,2\cdot q_2+1,1,i_1,0), & C_{10q_2+6+n} & = & (n,1,2,n-n,1,i_8,0), \\ C_{10q_2+3} & = & (0,1-1,1+1,n,1,i_2,0), & C_{10q_2+7+n} & = & (n,1,2,0,1,i_9,0), \\ C_{10q_2+4} & = & (0,0,1,2\cdot q_2,1,i_6,0), & C_{10q_2+8+n} & = & (n,1+1,2-1,0,1,i_9,0), \\ C_{10q_2+5} & = & (0,0+2,2,n,1-1,i_7,0), & C_{10q_2+9+n} & = & (n,2+1,1-1,0,1,i_9,0), \\ C_{10q_2+6} & = & (0,2-1,2,n,0+1,i_8,0), & C_{10q_2+10+n} & = & (n,2+1,0,0,1,i_1,0), \end{array}$$

In addition, bearing in mind that $r_2 = 1$, because n is an odd number, let us remember that $n = q_2\cdot 2 + r_2$ therefore, the number of transition steps is:

$$\begin{aligned} 10q_2 + 10 + n &= 4q_2 + \underbrace{6q_2}_{3\cdot 2q_2} + \underbrace{3}_{3r_2} + 7 + n = 4q_2 + \underbrace{3\cdot 2q_2 + 3r_2}_{3(q_2\cdot 2+r_2)} + 7 + n \\ &= 4q_2 + 3n + 7 + n = 4q_2 + 4n + 7 = \alpha_2, \end{aligned}$$

thus, the computation halts in $\alpha_2 = \beta_2$ steps, thus, the result is verified for $k = 2$.

Let k be such that $2 \leq k < p-1$ and let us assume that $\phi(k)$ is true. Let us see that it is also true for $k+1$. Indeed, by induction hypothesis we have

$$C_{\beta_k} = (n, k+1, 0, 0, 1, i_1, 0),$$

Here we observe that we have the hypothesis of the Lemma 3 for $x = \beta_k$ and $d = k+1$. Therefore, $\mu(q_{k+1})$ is true, that is

$$C_{\beta_k + q_{k+1}(3(k+1)+4)} = (n - q_{k+1}(k+1), k+1, 0, (k+1)q_{k1}, 1, i_1, 0),$$

Notice that $n - q_{k+1}(k+1) = r_{k+1}$ and $n = q_{k+1}(k+1) + r_{k+1}$. This follows

$$\begin{cases} C_{\beta_k+q_{k+1}(3(k+1)+4)+1} = (r_{k+1}, k+1-1, 1, (k+1)q_{k+1}, 1, i_2, 0), \\ C_{\beta_k+q_{k+1}(3(k+1)+4)+2} = (r_{k+1}-1, k+1-1, 1, (k+1)q_{k+1}+1, 1, i_1, 0), \\ \cdots \\ C_{\beta_k+q_{k+1}(3(k+1)+4)+2r_{k+1}} = \\ (r_{k+1}-r_{k+1}, (k+1)-r_{k+1}, r_{k+1}, (k+1)q_{k1}+r_{k+1}, 1, i_1, 0), \end{cases}$$

$$C_{\beta_k+q_{k+1}(3(k+1)+4)+2r_{k+1}+1} = (0, (k+1)-r_{k+1}-1, r_{k+1}+1, n, 1, i_2, 0),$$

$$C_{\beta_k+q_{k+1}(3(k+1)+4)+2r_{k+1}+2} = (0, (k+1)-r_{k+1}-1, r_{k+1}+1, n, 1, i_6, 0),$$

$$C_{\beta_k+q_{k+1}(3(k+1)+4)+2r_{k+1}+3} = (0, (k+1)-r_{k+1}+1, r_{k+1}+1, n, 0, i_7, 0),$$

$$C_{\beta_k+q_{k+1}(3(k+1)+4)+2r_{k+1}+4} = (0, (k+1)-r_{k+1}, r_{k+1}+1, n, 1, i_8, 0),$$

$$\begin{cases} C_{\beta_k+q_{k+1}(3(k+1)+4)+2r_{k+1}+4+1} = (1, (k+1)-r_{k+1}, r_{k+1}+1, n-1, 1, i_8, 0), \\ \cdots \\ C_{\beta_k+q_{k+1}(3(k+1)+4)+2r_{k+1}+4+n} = (n, (k+1)-r_{k+1}, r_{k+1}+1, n-n, 1, i_8, 0), \end{cases}$$

$$C_{\beta_k+q_{k+1}(3(k+1)+4)+2r_{k+1}+n+5} = (n, (k+1)-r_{k+1}, r_{k+1}+1, 0, 1, i_9, 0),$$

$$\begin{cases} C_{\beta_k+q_{k+1}(3(k+1)+4)+2r_{k+1}+n+5+1} = (n, (k+1)-r_{k+1}+1, r_{k+1}+1-1, 0, 1, i_9, 0), \\ \cdots \\ C_{\beta_k+q_{k+1}(3(k+1)+4)+2r_{k+1}+n+5+(r_{k+1}+1)} = \\ (n, (k+1)-r_{k+1}+r_{k+1}+1, r_{k+1}+1-(r_{k+1}+1), 0, 1, i_9, 0), \end{cases}$$

$$C_{\beta_k+\underbrace{q_{k+1}(3(k+1)+4)+3r_{k+1}+n+7}} = (n, (k+1)+1, 0, 0, 1, i_1, 0),$$

Let us see that those arithmetic operations results in α_{k+1}, let us remember that $n = q_{k+1}(k+1) + r_{k+1}$, the following equalities holds:

$$\begin{aligned} q_{k+1}(3(k+1)+4) + 3r_{k+1} + n + 7 &= 4q_{k+1} + \underbrace{3(k+1)q_{k+1} + 3r_{k+1}}_{3((k+1)q_{k+1}+r_{k+1})} + n + 7 \\ &= 4q_{k+1} + 3n + n + 7 \\ &= \alpha_{k+1} \end{aligned}$$

$$C_{\beta_{k+1}} = (n, (k+1)+1, 0, 0, 1, i_1, 0),$$

Therefore, the result is verified for $(k+1)$. □

From Theorem 4, the formula $\phi(p_1 - 1)$ true, that is

$$\phi(p-1) \equiv C_{\beta_{p-1}} = (n, (p_1 - 1) + 1, 0, 0, 1, i_1, 0).$$

The hypothesis of the Lemma 3 is verified, then $\mu_{p_1}(q_{p_1} - 1)$ is true, remember that $n = p_1 q_{p_1}$, that is

$$C_{\beta_{p_1-1}+(q_{p_1}-1)(3p_1+4)} = (n-p_1(q_{p_1}-1), p_1, 0, p_1(q_{p_1}-1), 1, i_1, 0),$$

$$\begin{cases} C_{\beta_{p_1-1}+(q_{p_1}-1)(3p_1+4)+1} = (n-p_1q_{p_1}+p_1, p_1-1, 1, p_1(q_{p_1}-1), 1, i_1, 0), \\ C_{\beta_{p_1-1}+(q_{p_1}-1)(3p_1+4)+2} = (p_1-1, p_1-1, 1, p_1(q_{p_1}-1)+1, 1, i_1, 0), \\ \dots \\ C_{\beta_{p_1-1}+(q_{p_1}-1)(3p_1+4)+2p_1} = (p_1-p_1, p_1-p_1, p_1, p_1(q_{p_1}-1)+p_1, 1, i_1, 0), \end{cases}$$

$$C_{\beta_{p_1-1}+(q_{p_1}-1)(3p_1+4)+2p_1+1} = (0, 0, p_1, p_1q_{p_1}-p_1+p_1, 1, i_3, 0),$$

$$C_{\beta_{p_1-1}+(q_{p_1}-1)(3p_1+4)+2p_1+2} = (0, 0, p_1, n, 1, i_5, 0),$$

$$\begin{cases} C_{\beta_{p_1-1}+(q_{p_1}-1)(3p_1+4)+2p_1+2+1} = (0, 0, p_1-1, n, 1, i_5, 1), \\ \dots \\ C_{\beta_{p_1-1}+(q_{p_1}-1)(3p_1+4)+2p_1+2+p_1} = (0, 0, p_1-p_1, n, 1, i_5, p_1), \end{cases}$$

$$C_{\beta_{p_1-1}+(q_{p_1}-1)(3p_1+4)+3p_1+3} = (0, 0, 0, n, 1, i_{10}, p_1),$$

$$C_{\beta_{p_1-1}+(q_{p_1}-1)(3p_1+4)+3p_1+4} = (0, 0, 0, n, 1, \#, p_1).$$

Finally, the computation halts in $\beta_{p_1-1}+q_{p_1}(3p_1+4)$ steps and the output is p_1, which is, exactly, the first prime factor of n.

Remark 3. *For n a prime number, we can consider as a particular case of n an odd number, in this case we have $n = p_1 \cdot q_{p_1}$, where $q_{p_1} = 1$ and $p_1 = n$. In addition, we can observe that the invariants of the Lemma 3 and the Theorem 4 are verified. In particular, $\phi(n-1)$ is true:*

$$\phi(n-1) \equiv C_{\beta_{n-1}} = (n, (n-1)+1, 0, 0, 1, i_1, 0)$$

which satisfies the following:

$$\begin{array}{lcl|lcl} C_{\beta_{n-1}+1} & = & (n, n-1, 1, 0, 1, i_2, 0) & C_{\beta_{n-1}+2n+2} & = & (0, 0, n, n, 1, i_5, 0) \\ C_{\beta_{n-1}+2} & = & (n-1, n-1, 1, 1, 1, i_1, 0) & C_{\beta_{n-1}+2n+2+1} & = & (0, 0, n-1, n, 1, i_5, 1) \\ & \dots & & & \dots & \\ C_{\beta_{n-1}+2n} & = & (n-n, n-n, n, n, 1, i_1, 0) & C_{\beta_{n-1}+2n+2+n} & = & (0, 0, n-n, n, 1, i_5, n) \\ C_{\beta_{n-1}+2n+1} & = & (0, 0, n, n, 1, i_3, 0) & C_{\beta_{n-1}+3n+3} & = & (0, 0, 0, n, 1, i_{10}, n) \end{array}$$

$$C_{\beta_{n-1}+3n+4} = (0, 0, 0, n, 1, \#, n)$$

Therefore, the computation halts in $\beta_{n-1}+3n+4$ steps and the output is n, which is, exactly; the least prime factor of n.

Remark 4. *Formal verification is also a useful tool for studying time computational complexity. The worst case for this device is when the input is a prime number, let us suppose that the input n is a prime number, by the Remark 3, the machine halts in $S(n) = \beta_{n-1}+3n+4$ transition steps, taking into account the fact that*

$$\begin{aligned} \beta_{n-1} &= \sum_{k=2}^{n-1} \alpha_k \\ &= \sum_{k=2}^{n-1} 4q_k + 4n + 7 \\ &= 4n(n-2) + 7(n-2) + \sum_{k=2}^{n-1} 4\lfloor \frac{n}{k} \rfloor \\ &= 4n^2 - 8n + 7n - 14 + 4\sum_{k=2}^{n-1} \lfloor \frac{n}{k} \rfloor \\ &\le 4n^2 - n - 14 + 4\sum_{k=2}^{n-1} \frac{n}{k} \\ &= 4n^2 - n - 14 + 4n\sum_{k=2}^{n-1} \frac{1}{k} \\ &\approx 4n^2 - n - 14 + 4n\int_2^{n-1} \frac{1}{x} dx \\ &= 4n^2 - n - 14 + 4n(\log(n-1) - \log(2)) \\ &= 4n^2 + 4n\log(\tfrac{n-1}{2}) - n - 14, \end{aligned}$$

We can conclude that the time computational complexity of this machine is of quadratic order $O(n^2)$ for 1-ary encoding.
